# Echocardiography and cardiorespiratory changes post ketofol or atracurium/ketofol on acepromazine-methadone premedicated dogs

**DOI:** 10.1038/s41598-025-06722-2

**Published:** 2025-06-25

**Authors:** Marwa Abass, Alshimaa Farag, Reham A. Fahmy

**Affiliations:** 1 Department of Surgery, Anesthesiology, and Radiology, Faculty of Veterinary Medicine, Mansoura University, Mansoura, 35516 Egypt; 2 Department of Internal Medicine, and Infectious Diseases, Faculty of Veterinary Medicine, Mansoura University, Mansoura, 35516 Egypt; 3 Fellow of Veterinary Surgery, Oncology Centre, Mansoura University, Mansoura, 35516 Egypt

**Keywords:** Atracurium, Ketamine, Propofol, Cardiorespiratory, M-mode, Dogs, Drug discovery, Zoology

## Abstract

Cardiovascular failure has been recognized as the predominant cause of perioperative mortality in small animals, particularly dogs. This study was designed to evaluate the effectiveness of adding intravenous atracurium to a ketofol infusion during anesthesia in dogs. Thirty male mongrel dogs were premedicated with an intramuscular injection containing 0.02 mg/kg of acepromazine and 0.2 mg/kg of methadone. Thirty minutes later, the dogs were equally and randomly divided into two groups (n = 15): the Ketofol Group (KFG), in which anesthesia was induced using IV administration of 0.5 ml/kg of ketofol, and the atracurium/ketofol Group (AKFG), in which anesthesia was induced using IV administration of 0.25 mg/kg of atracurium with 0.5 ml/kg of ketofol. Following intubation, anesthesia was maintained by a variable intravenous infusion at 0.2 ml/kg/min in KFG or a combination of 0.01 mg/kg/min atracurium and 0.2 ml/kg/min ketofol in AKFG. Respiratory frequency (fR), mean arterial pressure (MAP), heart rate (HR), oxygen saturation of hemoglobin (SpO_2_), end-tidal carbon dioxide concentration (EtCO_2_), rectal temperature (RT), the quality of induction, intubation, recovery period, ejection fraction percentage (EF%), fractional shortening percentage (FS%), and stroke volume (SV) were recorded. The ketofol doses were significantly lower, *P* ≤ 0.01, in the AKFG group (4.2 ± 0.44 mg/kg) than in the KFG group (2.27 ± 0.6 mg/kg). There were statistically significant increases in RR, HR, MAP, EtCO_2_, and echocardiography parameters in the AKFG group compared to the KFG group. Additionally, the AKFG group exhibited a significant reduction in induction, intubation, and recovery scores compared to the KFG group. Adding atracurium to ketofol during dog anesthesia positively impacts the hemodynamic and cardiac parameters and improves the quality of induction, intubation, and recovery.

## Introduction

Cardiovascular failure is the most frequently reported cause of perioperative death in dogs^1^. The mortality rate ranges from 30 to 70% and is primarily attributed to anesthetic overdose, myocardial depression, cardiac arrhythmias, circulatory failure, and hypovolemia^2–6^. In veterinary cardiology, the echocardiogram has recently become a crucial adjunctive examination^7–10^. With its non-invasive nature, wide accessibility, and ability to provide real-time imaging, this tool is essential for identifying anatomical structures, assessing the size and function of the heart and large vessels, evaluating hemodynamics, and detecting both congenital and acquired heart diseases^11,12^. M‐mode echocardiography determines standard reference values for dogs^13^ and other animals^14–18^.

Propofol, an alkyl phenol compound, exhibits a rapid onset and brief duration of action, rendering it primarily suitable for use as an induction agent^19,20^. However, its use as the sole anesthetic agent for prolonged surgical procedures is limited due to its dose-dependent respiratory depression and hypotension^19,20^. Another common anesthetic drug used in dogs is ketamine, a dissociative agent with a rapid onset of action and mild to moderate respiratory and cardiovascular effects^5,6^. It causes mild to moderate transient impacts on blood pressure, cardiac output, and heart rate, leading to convulsions, muscle stiffness, and ultimately to eventful recoveries^6^. The overall cardiovascular effects of propofol and the potential benefits of combining co-induction agents in dogs are still a subject of debate^21–23^.

Ketofol, which is a combination of propofol and ketamine, is extensively utilized for short procedural sedation and analgesia, so that it is used to achieve the hemodynamic stability observed when these drugs are separately administered while providing the convenience of a single infusion^20,24,25^. Ketofol (1:1) is frequently administered as a variable intravenous infusion to provide sedation and analgesia for both humans and animals^4,26–28^. Combining ketamine with muscle relaxants and tranquilizers can prolong the anesthetic effect, prevent seizures, and achieve analgesia and muscle relaxation^29^.

Neuromuscular blocking agents (NMBA) are commonly used in human or veterinary surgeries as neuromuscular block (NMB) allows easier surgical access to the abdominal cavity as well as organ manipulation^30^. The depth of NMB refers to the muscular response following stimulation of a motor nerve, typically the ulnar nerve. The contraction of the limb muscle is quantified by the train-of-four method (TOF) after TOF stimulation^31^.

In contrast, neuromuscular blockers (NMBs) do not induce central nervous system depression. Therefore, they are primarily administered along with anesthetics and analgesics during surgery. NMBs are affected by anesthetics, and their most prominent augmentation is observed when used in combination with hypnotic drugs such as propofol^32,33^. In dogs, the administration of low doses of atracurium (25, 50, or 75 μg/kg) results in muscle relaxation such as oculomotor, tail, and limbs without complete blocking the intercostal and diaphragmatic muscles in dogs. Hence, the severity of atracurium side effects is contingent upon the dosage and administration technique^34,35^.

To date, no previous studies have evaluated the equality of anesthesia, including induction, intubation, and recovery during a variable rate infusion (CRI) of atracurium/ketofol in healthy dogs. Therefore, this study was conducted to assess the impact of ketofol infusion anesthesia alone or in combination with atracurium on anesthesia quality during induction, intubation, and recovery. Besides, this study aimed to examine any changes in hemodynamic and echocardiographic parameters in dogs. We hypothesized that adding atracurium to ketofol would result in greater stability in cardiovascular parameters than ketofol alone in dogs.

## Materials and methods

### Animal ethics and consent

This study is in accordance with ARRIVE guidelines. All experiments conducted in this study were in strict accordance with relevant guidelines and regulations. This study had an approval number from the Faculty of Veterinary Medicine- Mansoura University Animal Care and Use Committee, Egypt, with the registration code R/146, and informed owner consent was obtained.

### Animals

Based on sample size calculations with an alpha error of 0.05, a beta error of 0.90, an effect size of 0.5, and a critical F of 4.196, this prospective study indicated that the total sample size was 30 dogs. Thus, each group consisted of 15 dogs.

Thirty male mixed-breed dogs were admitted to Mansoura University Veterinary Hospital. The dogs were brought from a shelter situated in Mansoura under the supervision of the Mansoura University Animal Care and Use Committee.

The dogs’ ages were 25 ± 3 months, and their weight was 24 ± 2.36 kg. All dogs were deemed healthy following a clinical examination classified as ASA I. The dogs were owned by a private shelter specifically designed and constructed to accommodate abandoned and stray dogs. The dogs were relocated to the Hospital of Veterinary Medicine and housed individually in cages with dimensions of 1 × 1.5 × 1.2 m. The dogs were kept in these cages until one day after the completion of the experiments. The dogs were confined indoors all day without any opportunity for outdoor physical activity, but they were allowed to be free from their cages for approximately 15–20 min during feeding time. The animals’ health status was assessed through a comprehensive physical, biochemical (urea, creatinine, glucose, albumin, alanine aminotransferase, Na^+^, Ca^++^, K^+^, and Mg^++^), and hematological examination. The exclusion criteria encompass evident inflammatory conditions, aggressive behavior, any hematologic crises in the complete blood count (CBC), and abnormal biochemical analysis results.

### Anaesthetic protocols

The anaesthesia drugs and infusion pumps used in this study were prepared before the study. All syringes were labeled with numbers by (RF). The anaesthesia protocol was conducted by a blind, experienced veterinarian (MA). The animals were fasted for a minimum of 6 h, with free access to water until pre-medication was administered. All dogs in this study were premedicated with 0.02 mg/kg of acepromazine maleate (Prequillan, 10 mg/ml, Fatro, Switzerland) and 0.2 mg/kg of methadone HCL (methadone 1%, Bioniche Pharma, USA) intramuscularly in the thigh muscles. After thirty minutes, a 20-gauge intravenous cannula (Mdk Mart, Egypt) was inserted into a cephalic vein. For non-invasive oscillometric blood pressure monitoring, a suitable blood pressure cuff (Critikon Soft-cuff, GE Healthcare, UK), with a cuff width/metatarsal circumference ratio of 0.3, was placed over the dorsomedial artery (M69S user’s manual, China).

The study design was investigated in a prospective, randomized, blind controlled trial, in which the premedicated dogs were assigned to two groups (n = 15) to receive an intravenous induction mixture. The first group (KFG) received 0.5 mL/kg of ketofol (1:1), a combination of 100 mg of propofol (Diprivan 10 mg/ml, AstraZeneca, Egypt), and 100 mg of ketamine (Ketamine 100 mg/ml; Alfasan, Holland) (i.e.: each 10 ml of propofol_1%_ added to 1 ml of ketamine_10%_; 0.5 ml of ketofol contains about 4.17 mg ketamine and 4.17 mg propofol). This admixture of ketofol was kept for a maximum of 12 h^28^. The second group (AKFG) IV received 0.25 mg/kg of atracurium (atracurium besylate, 10 mg/ml, Hameln, Germany) combined with 0.5 mL/kg of ketofol (both drugs were prepared in one syringe).

The quality of induction in dogs was evaluated^36^ (Table 1). The dogs that showed a rough (marked excitement, muscle twitching, paddling of limbs, head movement, or severe excitement with vocalization, ie, scoring 2 or 3) were excluded from this study. Two dogs in the KFG had a rough induction score of 3, and one in the AKFG had a rough induction score of 2. The endotracheal tube was connected to a rebreathing circle system, and the oxygen had adjustable flow rates from 3 L/minute.

**Table 1:** Description of scales for induction, intubation, and recovery in dogs^34–37^.

Sedation score		
No Sedation	Complete a ware animal	0
Slight Sedation	Almost normal; able to stand easily, but appears somewhat fatigued, subdued or somnolent	1
Moderate Sedation	Able to stand but prefers to be recumbent; sluggish; ataxic or uncoordinated	2
Profound Sedation	Unable to rise but can exhibit some awareness of environment; responds to stimuli through body movement; may be lateral or sternal recumbency	3
Unresponsive	In a state of coma or semi-coma from which little or no response can be elicited; remains in lateral recumbency	4
Intubation score		
Smooth/Excellent	Intubation successful in one attempt without physical reaction to intubation (no swallowing, coughing, tongue, or jaw movement)	0
Good	Intubation was successful in one attempt with a physical response to intubation (some tongue movement, slight cough)	1
Fair	Intubation successful after more than one attempt with or without physical response to intubation (marked tongue/jaw movement and swallowing or coughing)	2
Poor	Intubation impossible and requiring additional propofol dose and a second attempt at intubation	3
Induction score		
Smooth/Excellent	Without excitement	0
Good	Slight excitement, muscle twitching or movement of limbs	1
Fair	Marked excitement, muscle twitching, paddling of limbs, head movement	2
Poor	Severe excitement with vocalization	3
Recovery score		
Smooth/Excellent	Smooth, calm, uncomplicated	0
Good	Minimal vocalization and/or struggling	1
Fair	Moderate vocalization and/or struggling	2
Poor	Marked vocalization and/or struggling	3

Anesthesia was maintained immediately 10 min after induction throughout the study using a variable rate infusion system (VRI; Mindray SK-500II, China). The first group (KFG) received ketofol at a 0.2 mL/kg/minute dose. The second group (AKFG) received a combination of atracurium at a dose of 0.01 mg/kg/minute and ketofol at a dose of 0.2 mL/kg/minute (i.e., 3.3 mg/kg propofol and 3.3 mg/kg ketamine). The dogs in both groups (KFG or AKFG) were administered incremental doses of ketofol at 0.2 mL/kg only in case of withdrawal or twitching of the non-stimulated right limbs, head movement, chewing, licking, blinking, or swallowing occurred.

Both groups of dogs received lactated Ringer’s solution at a rate of 5 mL/kg/h IV. The variable rate infusion of ketofol, atracurium, and IV fluid therapy was disconnected after 50 min. Afterward, the dogs were allowed to recover and were extubated once they were able to swallow.

### Data collection and evaluation times

An observer who was blinded to the treatment groups assessed the quality (score) and time per minute of sedation, induction, intubation, and recovery^36–39^ (Table 1). Induction time was recorded as the time from the administration of ketofol or atracurium/ketofol (time 0 of anesthesia) until the dogs achieved recumbency. Intubation time was recorded as the time required to insert an endotracheal tube. Recovery time was recorded as the time from the stopped administration of VRI ketofol or atracurium/ketofol till the dogs stood.

The time (per second) to first breath after induction of anesthesia with ketofol or atracurium/ketofol and the total doses (ml) of ketofol used during the maintenance of anesthesia were recorded and analyzed.

Furthermore, the following parameters were assessed: the mean arterial blood pressure (MAP), heart rate (HR), respiratory frequency (fR), oxygen saturation of hemoglobin (SpO2), end-tidal carbon dioxide concentration (EtCO_2_), and rectal temperature (RT) were measured at previous time points using a multi-parameter monitor (Aisys Carestation, General Electric Co; Datex-Ohmeda, GE Healthcare Inc., Madison, WI, USA). Additionally, the echocardiographic examination was conducted using portable ultrasonographic equipment (Esaote MylabTM 30 Vet Gold, Brazil) with a sectoral transducer (PA240) at a frequency range of 1 to 4 MHz. These parameters were assessed at different time points: before the administration of pre-anesthetic medications considered as a baseline (M0); 30 min after the administration of pre-anesthetic medications (M1); immediately after intubation (M2); and at 10, 20, 30, 40, and 50 min after maintaining anesthesia (T10, T20, T30, T40, and T50).

The dogs were placed in right lateral recumbent positions, and echocardiographic measurements followed the guidelines outlined in the literature^40–45^. In the right parasternal window, on the transverse axis, the following parameters were evaluated using M mode: diastolic and systolic interventricular septum thickness (IVSd and IVSs cm), diastolic and systolic left ventricular internal diameter (LVIDd and LVIDs cm), left ventricular posterior wall thickness at end-diastole and end-systole (LVPWd and LVPWs cm), interventricular septal thickness at end-diastole and end-systole (IVSTd and IVSTs cm), left ventricular volume at end-diastole and end-systole (EDV and ESV ml). The stroke volume (SV ml), fractional shortening percentage (FS%), ejection fraction percentage (EF%), and the left atrial/aortic diameter ratio (La/Ao ratio) were calculated from the measurements performed in M mode.

### Statistical analysis

Data were analyzed using Minitab 16 Statistical Software (Minitab Ltd., UK). Deviations from assumptions of normality were assessed using graphical displays and the Kolmogorov–Smirnov test. Demographic data, induction, intubation, recovery score, and ketofol doses were compared using the Mann–Whitney U-test. The data were presented as median (range). Statistical significance was set at *P* < 0.05. The unpaired Student’s t-test was used to compare the average HR, fR, MAP, RT, SpO2, ETCO_2,_ and echocardiographic parameters at different time points between the groups.

Repeated measures analysis of variance (ANOVA) was used, followed by the Bonferroni test, to compare means within each group at time zero. To compare between groups at each time, one-way ANOVA was performed. The level of significance was set at *P* ≤ 0.05. The normally distributed data were presented as mean ± standard deviation, while non-normally distributed data were presented as median (range).

## Results

There were no significant differences between animals at the baseline (M0), and all animals were classified as ASA I. The sedation score following the administration of acepromazine and methadone in both groups was 2 (1–2), with no significant differences (*P* = 0.825). In addition, there was no difference between the groups following pre-anesthetic administration of acepromazine/methadone. None of the animals in either group received additional doses of sedation agents.

The induction time in the AKFG was significantly faster in comparison to the KFG (2.49 ± 0.31 min vs. 6.28 ± 0.27 min; *P* < 0.04). The intubation time did not show significant differences among the animals (the KFG was 4 ± 2 min vs. the AKFG was 5 ± 1 min; *P* = 0.782). Conversely, the recovery time was considerably longer in the KFG than in the AKFG (9.49 ± 0.3 vs. 5.49 ± 1.23 min; *P* = 0.011).

The total VRI dose of ketofol used for maintaining anesthesia in the KFG group was 4.2 ± 0.44 ml/kg, significantly higher than the dose used in the AKFG group (2.27 ± 0.6 ml/kg; *P* ≤ 0.05).

**Fig. 1:**
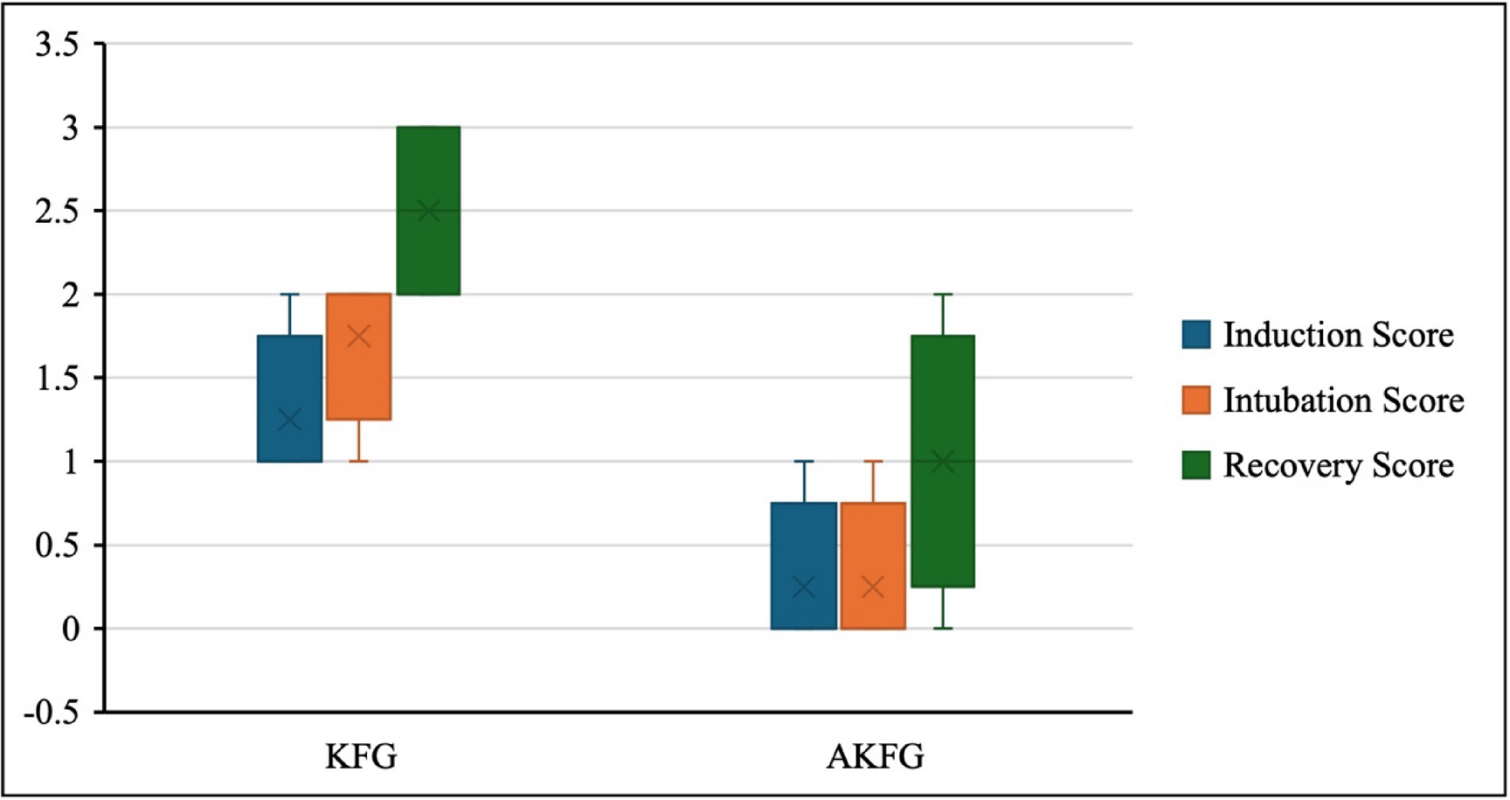
In dogs, Induction, intubation, and recovery scores during anesthesia with atracurium/ketofol (AKFG) and ketofol (KFG).

Furthermore, the induction, intubation, and recovery scores in the AKFG substantially increased and considerably improved compared to the KFG (0 (0–1), 1(0–1), and 1 (0–2) vs. 1(0–1), 2(1–2) and 2 (2–3); *P* = 0.04; Fig. 1). Overall, induction, intubation, and recovery were smooth in all dogs in AKFG. In contrast, the five dogs in the KFG group experienced muscle twitching during the induction period and were given additional induction doses of ketofol 0.5 ml/kg.

Following sedation (M1), all recording parameters significantly reduced compared to baseline (M0) without difference between groups. The rectal temperature in all animals remained between 37.2 and 37.9 °C without a significant difference at any time point.

**Fig. 2:**
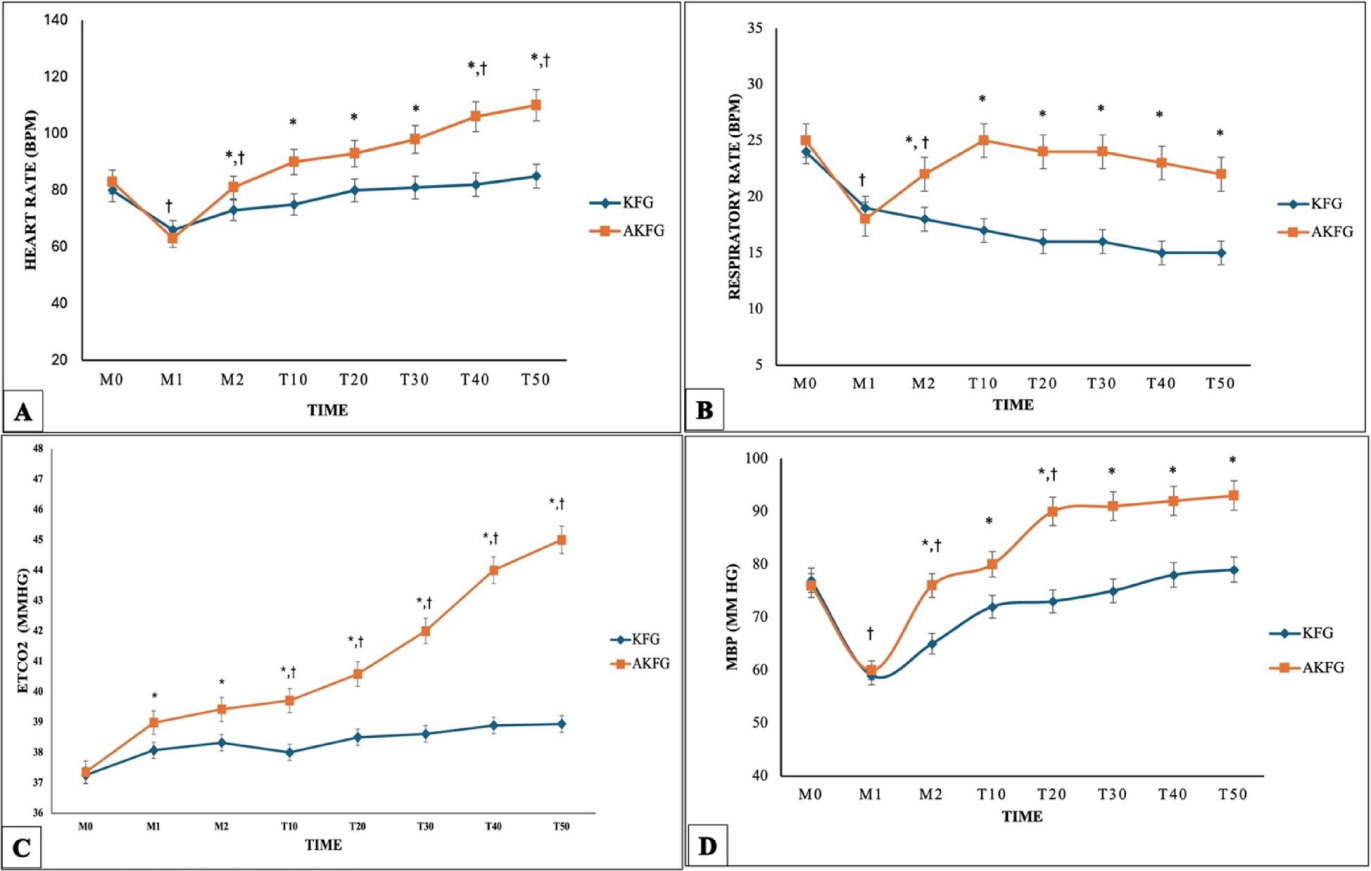
Hemodynamic parameters in dogs during anesthesia with atracurium/ketofol (AKFG) and ketofol (KFG). (**A**) Heart rate (HR bpm); (**B**) Respiratory frequency (fR bpm); (**C**) End-tidal carbon dioxide concentration (EtCO_2_ mmHg), and (**D**) Mean arterial blood pressure (MAP mmHg).

The heart rate was significantly higher in the AKFG (120 ± 10 bpm) than in the KFG (96 ± 9.1 bpm; *P* = 0.02) post-induction and intubation (M2) and till the end of the anesthesia (T50) in (Fig. 2A).

Time to the first spontaneous breath after induction of anesthesia with ketofol or atracurium/ketofol was substantially different between the KFG (6 ± 3 s) vs. the AKFG (2 ± 2 s; *P* = 0.043) post-induction. The KFG group experienced a more significant reduction in respiratory rate (fR) compared to the AKFG group (12 ± 1.23 bpm vs. 17 ± 2.54; *P* = 0.025) at M2 till T50 (Fig. 2B). Oxygen saturation of hemoglobin (SpO2) was significantly higher in the KFG group than in the AKFG group (98.47 ± 0.52 vs. 95.33 ± 0.61%; *P* = 0.036). Unlike End-tidal carbon dioxide level (EtCO_2_) significantly increased in the KFG group than the AKFG group at M2 (38.33 ± 1.21 vs. 39.47 ± 1.32 mmHg; *P* = 0.016) till T50 (Fig. 2C). Immediately after intubation (M2), the MAP was significantly higher in the AKFG compared to the KFG (89 ± 8 vs. 69 ± 5 mmHg; *P* = 0.033) and throughout the anaesthetic period T50 in (Fig. 2D).

The effects of acepromazine/methadone, ketofol, and atracurium/ketofol during the experimental period on the echocardiographic parameters between the groups and within the same group are presented (Table 2). Echocardiography findings of SV, EF%, and FS% were significantly affected by the time for ketofol (*P* = 0.012) and atracurium/ketofol (*P* = 0.031) compared with the post-sedation period M1. However, the AKFG recorded significantly higher values (*P* ≤ 0.05) of SV, EF%, and FS% at M2 compared with the KFG during the maintenance period of anesthesia.

**Table 2:** Mean ± SD Variation of different echocardiographic parameters in the KFG and AKFG groups at various time points in dogs.

Parameters	Time	M0	M1	M2	T10	T20	T30	T40	T50
	Group	Left ventricle parameters							
IVSd (cm)	KFG	1.18 ± 0.31	0.91 ± 0.41^†^	1.31 ± 0.02	1.35 ± 0.15	1.37 ± 0.08	1.52 ± 0.11	1.51 ± 0.21	1.51 ± 0.2
	AKFG	1.16 ± 0.22	0.85 ± 0.14^†^	1.66 ± 0.31*^,^^†^	1.79 ± 0.12*	1.90 ± 0.10*	1.99 ± 0.05*	2 ± 0.25*	2 ± 0.25*
IVSs (cm)	KFG	1.72 ± 0.71	0.78 ± 0.78^†^	2.94 ± 1.17	2.89 ± 1.44	2.97 ± 1.15	2.93 ± 0.55	2.72 ± 1.66	2.72 ± 1.6
	AKFG	1.75 ± 0.29	0.71 ± 0.35^†^	3.39 ± 1.31*^,^^†^	3.05 ± 1.12*	3.28 ± 1.41*	3.17 ± 1.02*	3.44 ± 1.90*	3.44 ± 1.9*
LVIDd (cm)	KFG	1.66 ± 0.32	0.92 ± 0.10^†^	2 ± 0.71	1.72 ± 0.12	2.22 ± 0.08	2.66 ± 0.17	2.39 ± 0.10	2.39 ± 0.1
	AKFG	1.63 ± 0.36	0.95 ± 0.50^†^	2.34 ± 0.14*^,^^†^	2.72 ± 0.83	2.72 ± 0.33	2.98 ± 0.89*	2.90 ± 0.16*	2.90 ± 0.1*
LVIDs (cm)	KFG	1.42 ± 0.17	0.93 ± 0.03^†^	1.17 ± 0.83	1.33 ± 1.17	1.66 ± 1.55	1.61 ± 1.44	1.83 ± 1.44	1.83 ± 1.4
	AKFG	1.39 ± 0.31	0.89 ± 0.13^†^	1.33 ± 0.56*^,^^†^	2 ± 1.02*	1.66 ± 1.55*	2.15 ± 1.60*	2.22 ± 1.21*	2.22 ± 1.1*
LVPWd (cm)	KFG	1.22 ± 0.72	0.85 ± 0.67^†^	2.61 ± 0.89	2.33 ± 1.61	1.89 ± 1.44	1.89 ± 1.17	2.44 ± 1.77	2.44 ± 1.7
	AKFG	1.39 ± 0.89	0.81 ± 0.09^†^	3.11 ± 1.61*^,^^†^	2.89 ± 1.24*	2.16 ± 1.90*	2.50 ± 1.10*	2.72 ± 0.91*	2.72 ± 0.1*
LVPWs (cm)	KFG	1.67 ± 1.22	0.82 ± 1.06^†^	2.39 ± 1.94	2.3 ± 1.01	1.66 ± 0.50	1.44 ± 1.28	1.89 ± 1.44	1.89 ± 1.4
	AKFG	1.54 ± 1.34	0.87 ± 0.06^†^	2.44 ± 1.23*^,^^†^	2.39 ± 0.9*	2.50 ± 1.11*	2.22 ± 1.21*	2.72 ± 1.01*	2.72 ± 1.1*
EDV (ml)	KFG	35.61 ± 3.71	10.94 ± 5.09^†^	13.97 ± 3.76	15 .14 ± 1.12	18.14 ± 5.40	20.90 ± 5.60	25.59 ± 5.60	25.1 ± 5.1
	AKFG	32.37 ± 5.12	11.10 ± 2.05^†^	21.36 ± 3.21*^,^^†^	23.99 ± 1.93*	24.92 ± 2.36*	25.03 ± 5.01*	27.65 ± 2.56*	27.1 ± 2.1*
ESV (ml)	KFG	14.82 ± 1.58	6.67 ± 0.58^†^	7.97 ± 1.15	8.36 ± 0.31	9.61 ± 1.75	10.17 ± 0.17	10.29 ± 0.04	10.3 ± 0.1
	AKFG	13.29 ± 1.43	6.17 ± 0.58^†^	8.97 ± 1.30*^,^^†^	13.36 ± 1.1*	12.61 ± 0.09*	12.17 ± 1.57*	13.94 ± 1.32*	14.1 ± 1.3*
SV (ml)	KFG	11.03 ± 0.93	2.51 ± 1.90^†^	3.39 ± 1.42	3.78 ± 0.42	3.17 ± 0.05	5.90 ± 0.43	7.71 ± 0.60	7.8 ± 0.60
	AKFG	11.93 ± 0.38	2.93 ± 0.45^†^	4.61 ± 1.12*^,^^†^	5.51 ± 1.73*	6.32 ± 0.91*	7.46 ± 0.92*	8.45 ± 1.11*	8.5 ± 1.11*
EF (%)	KFG	64 ± 2.1	55.2 ± 3.21^†^	59 ± 4.32	60 ± 4.27	61 ± 4.17	60 ± 2.75	64 ± 5.75	64 ± 5.75
	AKFG	65 ± 2.1	53.7 ± 2.88^†^	65 ± 5.60*^,^^†^	66 ± 3.39*	68 ± 5.34*	68 ± 7.36*	68 ± 4.6*	68 ± 4.6*
FS (%)	KFG	31 ± 2.12	25 ± 5.41^†^	38 ± 1.9	37 ± 1.79	35 ± 3.6	36 ± 4.25	36 ± 3.18	35 ± 3.18
	AKFG	30.81 ± 2.1	22.6 ± 3.52^†^	48 ± 4.9*^,^^†^	50 ± 0.99*	50 ± 2.78*	51 ± 2.12*	52 ± 2.10*	53 ± 2.10*
La/Ao ratio	KFG	1.72 ± 0.62	1.38 ± 0.18^†^	0.83 ± 0.08	1.01 ± 0.11	1.15 ± 0.50	1.28 ± 0.28	0.83 ± 0.11	0.83 ± 0.11
	AKFG	1.72 ± 0.12	1.36 ± 0.20^†^	0.72 ± 0.12*^,^^†^	1.78 ± 0.31*	1.83 ± 0.21*	1.72 ± 0.23*	1.82 ± 0.12*	1.92 ± 0.12*

*IVSd* interventricular septal thickness at end-diastole, *IVSTs* interventricular septal thickness at end-systole *LVIDd* left ventricular internal diameter at end-diastole, *LVIDs* left ventricular internal diameter at end-systole, *LVPWd* left ventricular posterior wall thickness at end-diastole, *LVPWs* left ventricular posterior wall thickness at end-systole, *EDV* left ventricular volume at end-diastole, *ESV* left ventricular volume at end-systole, *SV* stroke volume, *EF%* ejection fraction, *FS%* fractional shortening. Thirty minutes before (Baseline; M0), and after sedation with acepromazine/methadone (M1), and immediately after intubation (M2), 10, 20, 30, 40, and 50 min after maintenance with VRI KF or AKF (T10, T20, T30, T40, and T50).

*Significant P-value represents the comparison between groups. ^†^Significant P-value the comparison between the base line M0 at (*P* ≤ 0.05).

**Fig. 3:**
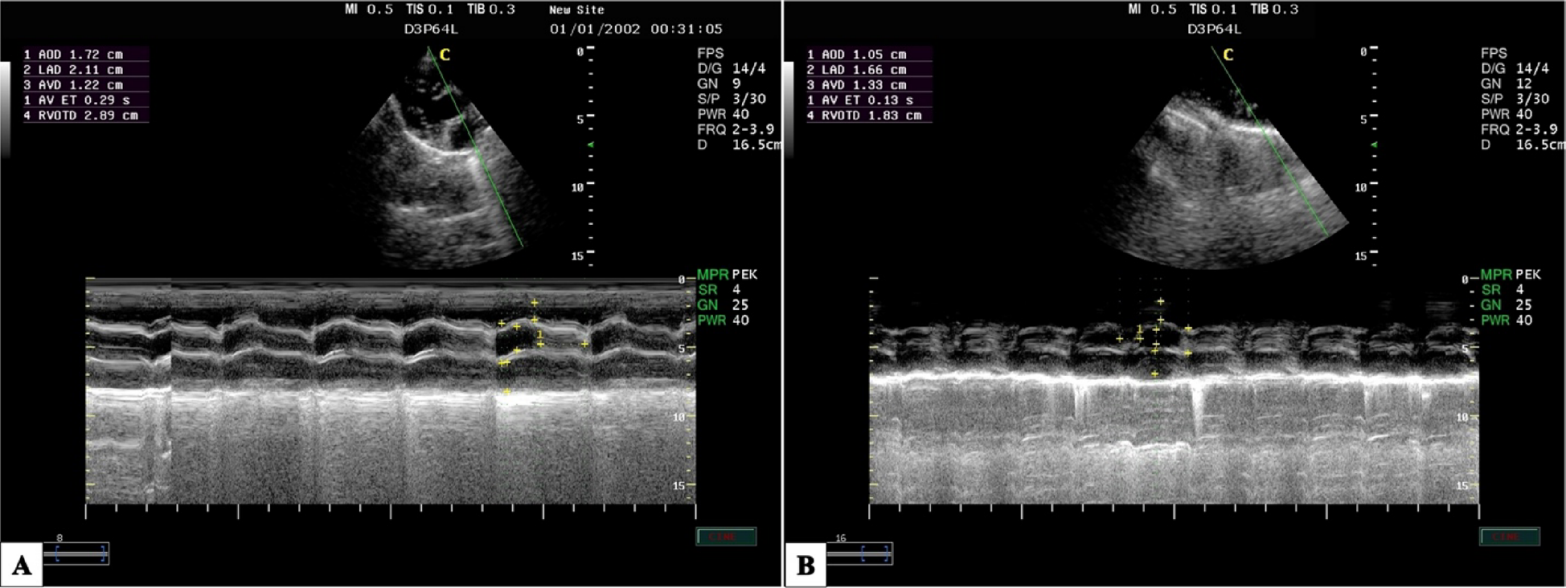
The right parasternal long-axis image of the left ventricle outflow tract in dogs using M-mode echocardiography during anesthesia with (**A**) atracurium/ketofol group (AKFG) and (**B**) ketofol group (KFG).

At M1, the La/Ao ratio in both groups exhibited a significantly reduced value (*P* = 0.01), which subsequently increased following induction and throughout anesthesia. Nevertheless, the AKFG values substantially increased compared to the KFG values (*P* ≤ 0.05; Fig. 3).

**Fig. 4:**
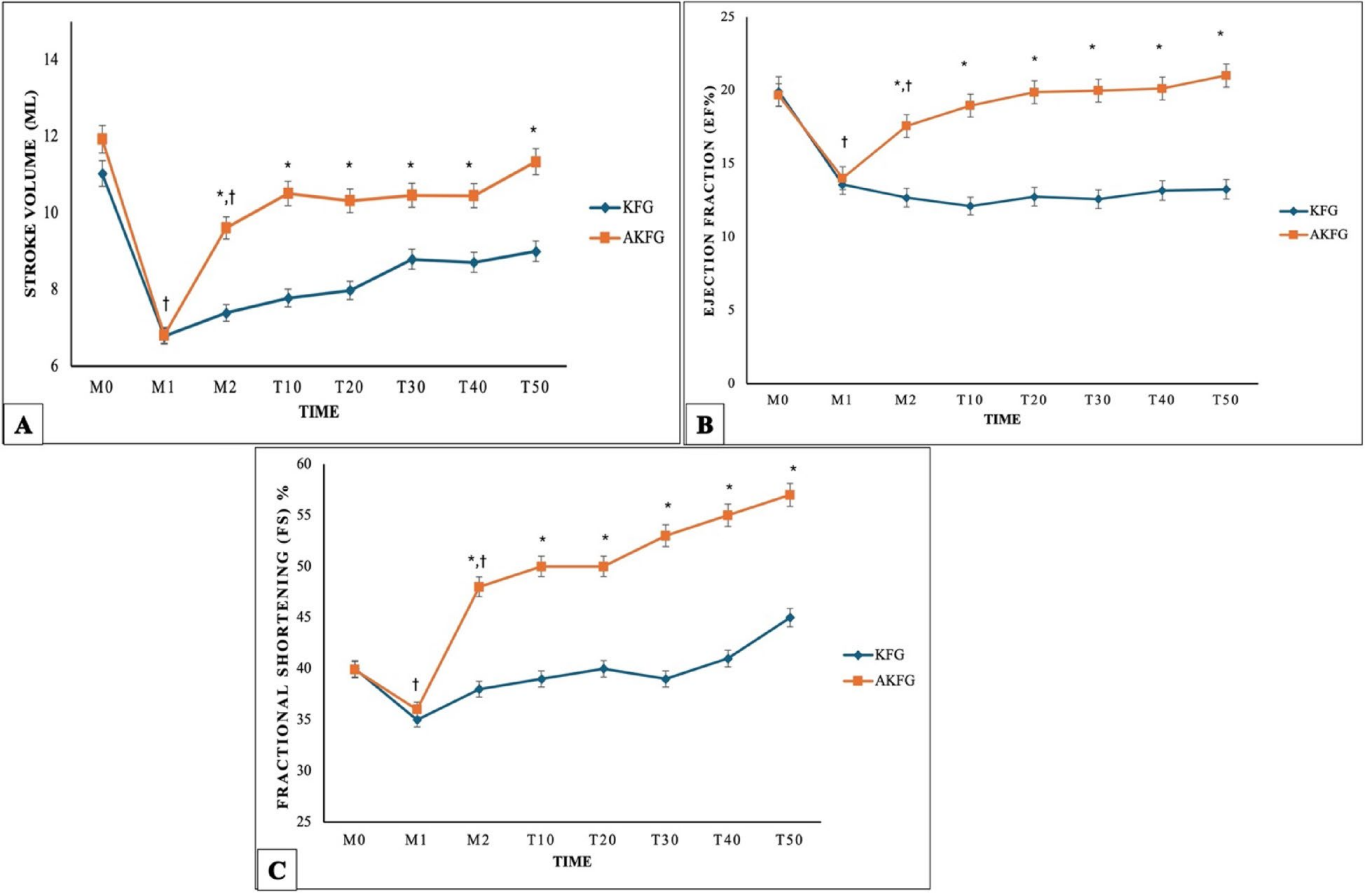
Echocardiographic parameters. (**A**) Stroke volume (SV ml); (**B**) Ejection fraction (EF%); (**C**) Fractional shortening during anesthesia with atracurium/ketofol (AKFG) and ketofol (KFG) in dogs.

The stroke volume (SV), ejection fraction (EF%), and fractional shortening (FS%) were significantly affected by time (*P* < 0.002). From M2 to T50, treatment with AKFG resulted in significantly higher values of SV ml (Fig. 4A), EF% (Fig. 4B), and FS% (Fig. 4C) compared to KFG.

**Fig. 5:**
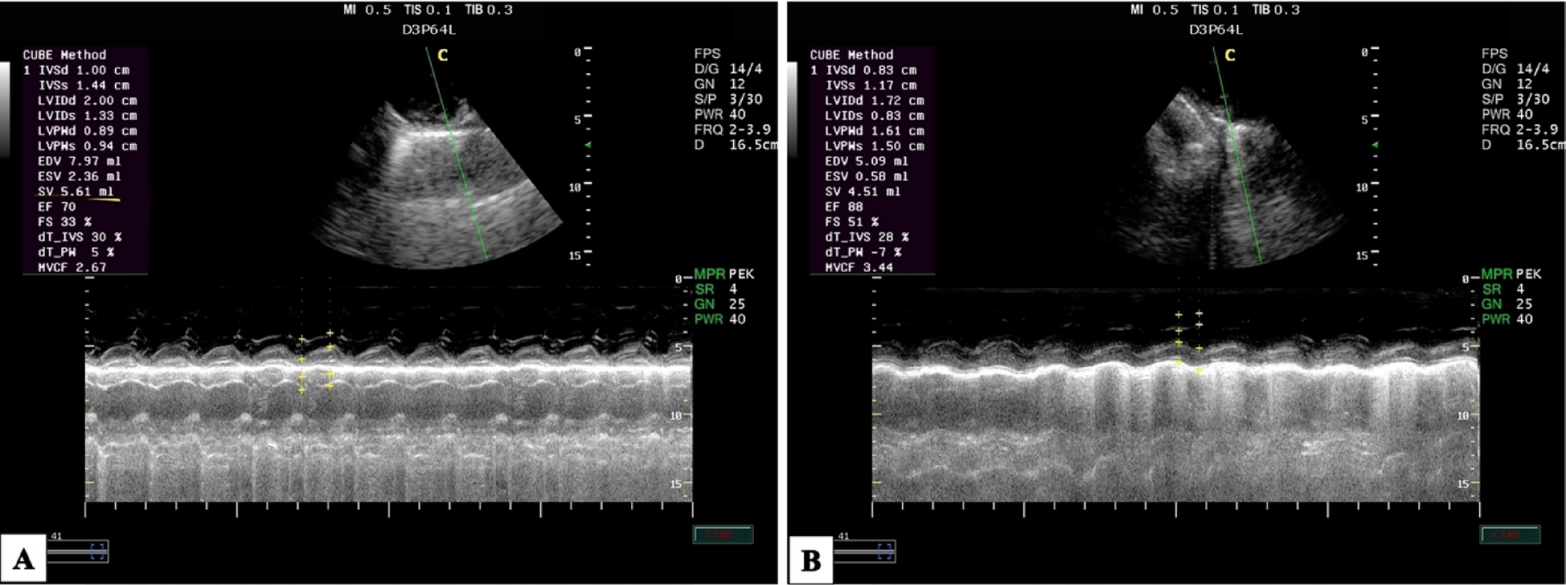
The right parasternal long-axis image of the left ventricle outflow tract view of the aortic valve in dogs using M-mode echocardiography during anesthesia with (**A**) atracurium/ketofol group (AKFG) and (**B**) ketofol group (KFG).

**Fig. 6:**
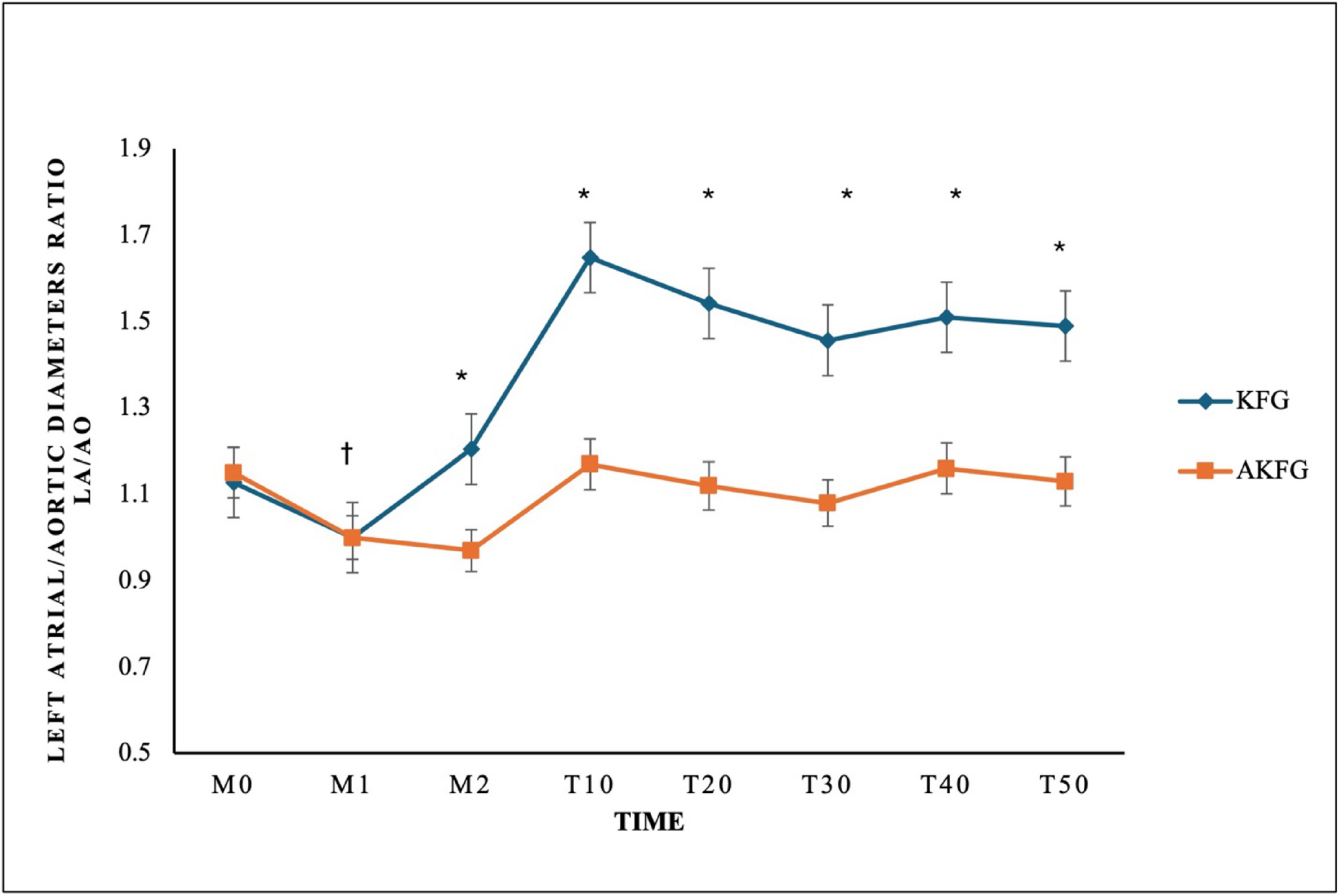
Left atrial/aortic diameter ratio (La/Ao ratio) during anesthesia with atracurium/ketofol (AKFG) and ketofol (KFG) in dogs.

In addition, the left atrial/aortic diameter ratio (La/Ao) showed a significant increase over time (*P* = 0.03) in both groups (Fig. 5). The AKFG group exhibited a significant increase in La/Ao (*P* = 0.01) at M2, T10, T20, T30, T40, and T50 compared to the KFG group (Fig. 6).

## Discussion

Previous studies have examined the use of ketamine in combination with propofol to attenuate the dose-dependent adverse events of propofol, including respiratory depression and hypotension^4,28,46,47^. Moreover, while the CV effects of ketofol treatment were satisfactory, respiratory adverse events did not decrease and tended to worsen as the ketamine ratio increased. In contrast, some studies have emphasized that ketofol, with a lower proportion of ketamine compared to propofol, may serve as a viable alternative to propofol alone, particularly in cases where there is a higher risk of respiratory depression^48,49^. No cardiovascular effects, such as impaired cardiac function or myocardial injury, were detected in healthy dogs when ketamine was administered at doses of 0.02 and 0.04 mcg/kg/minute for an extended time. In addition, the lower dosage that was examined exhibited enhanced stability in cardiac function, as evidenced by fewer changes in variables such as CI, MAP, HR, and EF compared to the baseline values^43^. Conversely, bolus doses of ketamine have a more significant effect on the CVS, leading to unfavorable conditions like hypertension and tachycardia^50^. The present research was designed to study the impact of ketofol 1:1 when added to atracurium on the heart rate, mean blood pressure, respiratory rate, SpO2, ETCO2, stroke volume, ejection fraction, and fractional shortening.

The findings of our study indicated that the administration of a VRI of 0.01 mg/kg/h of atracurium combined with 0.2 ml/kg/h of ketofol resulted in significantly higher HR, fR, and MAP compared to the administration of 0.2 ml/kg/h of ketofol alone. This finding aligns with a previous study^28^ that noted differences in delivery speed, and the possibility of dogs having a particular vulnerability to ketamine, should not be ignored.

The current study only evaluated respiratory effects by measuring fR, ETCO2, and SpO2, which coincides with^28^. However, additional information, such as blood gases and minute volume, would have been beneficial in evaluating respiratory function. Hypercapnia recorded in AKFG may be the cause of higher HR, RR, as an increase in carbon dioxide levels stimulates the sympathetic system, leading to tachycardia and tachypnea^51^.

In the current study, two dogs in the KFG had a rough induction score of 3, and one in the AKFG had a rough induction score of 2, were excluded to avoid an additional bolus and the effect of induction drugs (ketofol and atracurium).

AKFG demonstrated enhanced efficacy in tracheal intubation and anesthesia induction compared to KFG alone. These results are consistent with a study conducted^52^ that found that NMBs improved tracheal intubation.

However, Lerche et al. mentioned that^53^ the administration of rapid bolus ketamine with midazolam can lead to a transient decrease in ventilation in dogs. In our study, the KFG resulted in a more significant decrease in the rate of respiration than the AKFG. This result is inconsistent with a previous study^54^ indicating that the addition of ketamine to propofol did not exacerbate apnea when compared to propofol alone. In contrast to a study conducted in cats^55^, ketofol has been shown to cause no or minimal respiratory depression when baseline values were compared to post-induction measurements. The variation in the use of anesthetic agents was elucidated by^56^ those who mentioned that various factors determine the use of anesthetic agents. These factors include the surgical operation settings, the patient’s response to the anesthetic drug action, the animal species or breed, and the operated animal’s health status.

Ketamine can be used as a standalone anesthetic agent; however, it causes muscle rigidity, catalepsy, tachycardia, poor muscle relaxation, and hypertension. Therefore, the utilization of ketamine as a solitary anesthetic medication has been restricted to cases involving myoclonus and muscle hypertonicity, infrequent convulsions, and violent recovery. In addition, ketamine must be integrated with other sedatives, such as acepromazine and midazolam, to decrease adverse impacts^57–59^ such as muscle relaxation.

The current study found that the combination of acepromazine and methadone resulted in a level of sedation ranging from mild to moderate. This sedation was effective in allowing the placement of a catheter and facilitating the induction of anesthesia, eliminating the requirement for supplementary sedation. Consequently, there was a decrease in the negative impact on the cardiorespiratory function^60–62^. Acepromazine’s fundamental mechanism does not involve any analgesic effect, but it does have a prolonged duration of action. In addition, it is associated with hypotension due to notable peripheral vasodilatation and variability in its response in dogs^63^.

Sedation may be necessary when animals experience stress and echocardiographic examination becomes challenging^64^. Another study found that using a combination of acepromazine and hydromorphone for sedation does not impact M-mode and two-dimensional (2D) measurements despite the mild increase in HR^13,65^.

Specific drugs, such as ketamine and xylazine, can significantly decrease many echocardiographic variables, including the left ventricular lumen diameter in the diastolic phase, the fractional shortening, and the left atrium diameter (LA)^14^. Prior research revealed that propofol caused a mild decrease in systolic mitral annular velocity, but it did not affect diastolic function in Maine coon cats with hypertrophic cardiomyopathy (HCM)^66^. Nevertheless, the potential adverse cardiac effects of ketofol have not been fully elucidated. Furthermore, ketofol has been reported to provide smooth recoveries in other species, as revealed by^46,55^.

Atracurium is reported to decrease blood pressure due to histamine or prostacyclin release occasionally^20^. Other studies in dogs have not shown significant changes in cardiovascular parameters, blood pressure, and heart rate associated with administering atracurium followed by propofol induction^67,68^. Considering that TIVA needs a higher dose of NMBs than inhalation anesthesia^69^, using NMBs reduces spasmodic breaths during mechanical ventilation of the lungs and the plasma clearance process^70^. These advantages may cause hemodynamic stability and improve induction, intubation, and recovery scores registered in the AKFG^32,33^.

Contrary to specific induction anesthetics like alfaxalone, which have a more significant impact on body temperature, especially in the early stages of sedation^42^. The findings indicated a marginal and statistically insignificant decline in body temperature (approximately 1 °C) in both groups, a common occurrence during anesthesia.

Researchers have focused on standardizing M-mode echocardiography values according to the breed type. Multiple studies presented proposed values. Kawahara et al.^71^ found that the cardiac functional parameters, including EF%, CI, FS%, and SV, are commonly used to examine animal cardiac and hemodynamic function profiles. Anesthetic drugs have a direct and indirect effect on hemodynamics and cardiac performance, making them significant factors to consider in interpreting changes in these parameters under different pathophysiological and physiological circumstances^45,71,72^.

The decrease in HR improved the alignment of the M-mode echocardiographic measurements. Additionally, the decreased voluntary movements of the animals, due to the reassurance during sedation, aided the examination process^73^.

Consistent with^38^, left ventricular end-diastolic diameter (LVDd) and left ventricular end-systolic diameter (LVDs), as well as the other parameters, were significantly decreased after sedation with acepromazine/methadone compared to the baseline values. The decrease in LVDd and LVDs can be attributed to the hypotensive effects induced by the combination of acepromazine and methadone. Moreover, this reduction in diameter is sufficient to result in a decrease in the left ventricular volume and aortic diameters. When comparing the KFG to those in the baseline measurements, statistically significantly lower EF% and FS% were reported. The hypotensive effects of acepromazine, methadone, and propofol administration may be the cause. In the case of reduced blood flow to the heart, the passive pathway of blood from the left atrium to the left ventricle remains relatively constant, which may result in a smaller blood volume available for the active pathway. Therefore, it may lead to an increase in these parameters^41^.

The cardiac parameters, such as FS%, EF%, and SV, exhibited significant changes until the end of the anaesthetic period (*P* ≤ 0.05) in dogs that received the AKFG compared to the KFG. The most significant difference between the M1 and T10 was observed when atracurium and ketamine were administered via bolus injection. Interestingly, the wall thickness did not rapidly increase, suggesting that ventricular myocardial fibers in postnatal dogs tend to elongate more than they widen. Another possibility is that myocardial density increases with age while extracellular water concentration decreases. In addition, age had a significant impact on the mean values of left ventricular posterior wall thickness (LVPWs) and interventricular septal thickness at end-diastole (IVSd)^74^.

The La/Ao ratio depends on two variables: the diameter of the left atrium divided by the diameter of the aortic annulus^11^. In the present study, there was a significant reduction in the La/Ao ratio post-sedation with acepromazine/methadone, resulting from blood reduction. Subsequently, there was a rapid rise in the ratio after the administration of ketamine in the induction protocol, observed in both groups. However, the increase in the cardiac index was particularly pronounced in the AKFG due to the positive inotropic effect of the NMBs^75^ and the use of low doses of atracurium.

One limitation of the current study was the absence of controlled ventilation and monitoring with Train of Four. Further study should be made to evaluate the impact of this protocol when mechanical ventilation is used.

## Conclusion

It was noted that adding atracurium to Ketofol resulted in reduced duration of apnea after induction, as well as improved the quality of induction, intubation, and recovery periods. It had minimal effects on heart rate, respiratory rate, and blood pressure levels. Additionally, it can conserve echocardiographic parameters and cardiac hemodynamics. Consequently, it could be considered an alternative to ketofol for the induction and maintenance of anesthesia in healthy dogs.

## Data Availability

Data is provided within the manuscript.

## Abbreviations

IVSd: Interventricular septum thickness at diastole
IVSs: Interventricular septum thickness at systole
LVIDd: Left ventricular internal diameter at diastole
LVIDs: Left ventricular internal diameter at systole
LVPWd: Left ventricular posterior wall thickness at end-diastole
LVPWs: Left ventricular posterior wall thickness at end-systole
IVSTd: Interventricular septal thickness at end-diastole
IVSTs: Interventricular septal thickness at end-systole
EDV: Left ventricular volume at end-diastole
ESV: Left ventricular volume at end-systole.
SV: Stroke volume.
FS%: Fractional shortening percentage.
EF%: Ejection fraction percentage.
La/Ao: Left atrial/aortic diameter ratio
